# Evaluation of Petrifilm™ Select *E. coli *Count Plate medium to discriminate antimicrobial resistant *Escherichia coli*

**DOI:** 10.1186/1751-0147-50-38

**Published:** 2008-09-25

**Authors:** Shuyu Wu, Eirini Chouliara, Lars Bogø Jensen, Anders Dalsgaard

**Affiliations:** 1 Research Group of Antimicrobial Resistance and Molecular Epidemiology, Department of Microbiology and Risk Assessment, National Food Institute, Technical University of Denmark, Bülowsvej 27, DK-1790 Copenhagen V, Denmark; 2 Department of Veterinary Pathobiology, Faculty of Life Sciences, University of Copenhagen, Grønnegårdsvej 15, DK-1870 Frederksberg C, Denmark

## Abstract

**Background:**

Screening and enumeration of antimicrobial resistant *Escherichia coli *directly from samples is needed to identify emerging resistant clones and obtain quantitative data for risk assessment. Aim of this study was to evaluate the performance of 3M™ Petrifilm™ Select *E. coli *Count Plate (SEC plate) supplemented with antimicrobials to discriminate antimicrobial-resistant and non-resistant *E. coli*.

**Method:**

A range of *E. coli *isolates were tested by agar dilution method comparing the Minimal Inhibitory Concentration (MIC) for eight antimicrobials obtained by Mueller-Hinton II agar, MacConkey agar and SEC plates. Kappa statistics was used to assess the levels of agreement when classifying strains as resistant, intermediate or susceptible.

**Results:**

SEC plate showed that 74% of all strains agreed within ± 1 log_2 _dilution when comparing MICs with Mueller-Hinton II media. High agreement levels were found for gentamicin, ampicillin, chloramphenicol and cefotaxime, resulting in a kappa value of 0.9 and 100% agreement within ± 1 log_2 _dilution. Significant variances were observed for oxytetracycline and sulphamethoxazole. Further tests showed that the observed discrepancy in classification of susceptibility to oxytetracycline by the two media could be overcome when a plate-dependent breakpoint of 64 mg/L was used for SEC plates. For sulphamethoxazole, SEC plates provided unacceptably high MICs.

**Conclusion:**

SEC plates showed good agreement with Mueller-Hinton II agar in MIC studies and can be used to screen and discriminate resistant *E. coli *for ampicillin, cephalothin, streptomycin, chloramphenicol, cefotaxime and gentamicin using CLSI standardized breakpoints, but not for sulphamethoxazole. SEC plates can also be used to discriminate oxytetracycline-resistant *E. coli *if a plate-dependent breakpoint value of 64 mg/L is used.

## Background

*Escherichia coli *is the most common intestinal bacterium of the *Enterobacteriacae *family and its presence outside the intestine is often used as an indicator of faecal pollution and in surveillance programs of antimicrobial resistance. Screening and enumeration of antimicrobial resistant *E. coli *directly from samples, e.g., food or animal specimens, is needed to identify emerging resistance clones and to obtain quantitative data for epidemiological investigations [1]. MacConkey agar has been widely used as selective medium for isolation and enumeration of *E. coli *due to its low cost and high selectivity [2-4]. However, further identification of isolates is necessary since many species of *Enterobacteriaceae *can grow on MacConkey agar and colony characteristics are not sufficient to correctly identify *E. coli *[5]. More recently Petrifilm™ system (3M Microbiology Products, St. Paul., MN, USA) was developed for direct enumeration of *E. coli *[6]. The 3M™ Petrifilm™ Select *E. coli *Count Plate (SEC plate) is a sample-ready culture medium system consisting of plastic film with grids that are coated with selective agents, nutrients and gelling agent. Gel contains a β-glucuronidase indicator for confirmed detection of *E. coli *[7]. Only *E. coli *colonies are conspicuous on SEC plates, eliminating need for colony identification and allowing for a direct enumeration of *E. coli*. SEC plates have undergone some validation for enumeration of *E. coli *in food and water samples [8,9]. However, no previous studies have tested the feasibility of using SEC plates to discriminate antimicrobial-resistant *E. coli*. The main objective of this study was therefore to evaluate the SEC plate supplemented with antimicrobials to discriminate antimicrobial-resistant and non-resistant *E. coli *in a comparison study between SEC plate and Mueller-Hinton II agar, the standard medium commonly used for antimicrobial susceptibility testing in *E. coli*, and for which international recognized breakpoints and cut-off values for antimicrobial resistance have been defined [10].

## Methods

### Strain collection

Between 14 and 22 non-related *E. coli *strains were purposively selected based on their Minimal Inhibitory Concentration (MIC) values to eight selected antimicrobials as established in The Danish Integrated Antimicrobial Resistance Monitoring and Research Programme (DANMAP) strain database 2001–2005. The variable number of isolates reflected the available strains within the selected MIC ranges for the tested antimicrobials. These isolates represent a broad range of MICs allowing a better comparison between selected media. All strains were isolated from pigs or cattle and their MICs were originally determined in an automated microbroth dilution system (Sensititre; Trek Diagnostic Systems, West Sussex, UK).

Due to poor correlation of MIC values obtained by SEC plates and Mueller-Hinton II agar (BD Diagnostics, Sparks, MD, USA) for oxytetracycline, an additional 20 *E. coli *strains were randomly selected from the DANMAP strain database to confirm that a plate-dependent breakpoint of 64 mg/L can be used to differentiate oxytetracycline-resistant from -sensitive *E. coli*. Eight of these strains were determined as susceptible (MIC = 2–4 mg/L) and the remaining 12 were resistant (MIC = 16–32 mg/L) when using Mueller-Hinton II agar and CLSI established breakpoints [11].

### Antimicrobials and media used for susceptibility testing

Eight antimicrobials representing seven major classes of antimicrobials were selected for MIC testing, including: ampicillin, oxytetracycline, cephalothin, streptomycin, chloramphenicol, cefotaxime, gentamicin, and sulfamethoxazole (Sigma Chemical Co, St. Louis, Mo. USA) (Table 1). Stock solutions of 32 mg/ml were prepared for all antimicrobial agents prior to use according to CLSI guideline [10]. Mueller-Hinton II and MacConkey agar (Oxoid, Basingstoke, UK) were included in the MIC studies in comparison with SEC plates, as Mueller-Hinton II agar is standard medium used in antimicrobial susceptibility testing, and MacConkey agar is commonly used as selective medium for isolation of *Enterobacteriaceae*, including *E. coli*.

**Table 1:** Concentrations of antimicrobial agents and interpretative breakpoint or cut-off values.

Antimicrobial agents	Concentration ranges used in the three media (mg/L)	Quality control range^a ^for *E. coli *ATCC 25922 (mg/L)	CLSI clinical breakpoint (mg/L)			EUCAST epidemiological cut-offs (mg/L)
		
			S	I	R	R
Ampicillin	0.25–128	2–8	≤ 8	16	≥32	> 8
Cephalothin	2–64	4–16	≤ 8	16	≥32	> 32
Chloramphenicol	1–128	2–8	≤ 8	16	≥32	> 16
Gentamycin	0.5–64	0.25–1	≤ 2	4	≥8	> 2
Streptomycin	2–128	4–16^b^	≤ 8	16	≥32	> 16
Cefotaxime	0.06–128	0.03–0.12	≤ 8	16–32	≥64	> 0.25
Sulfamethoxazole	8–1024	8–32	≤ 256	_	≥ 512	> 256
Oxytetracycline	0.25–256	0.5–2	≤ 4	8	≥16	> 8

S, susceptible; I, intermediate; R, resistant.

^a ^CLSI standard for agar dilution susceptibility testing[11].

^b ^quality control range developed by the manufacturer Sensititre^®^

### Susceptibility testing (MIC study)

MIC studies were performed as described in CLSI guideline for Mueller-Hinton II and MacConkey agar plates [10]. In brief, a serial of twofold dilutions of tested antimicrobial were mixed with melted agar at 50°C in petri dishes with final concentrations of the antimicrobial as shown in Table 1. Inocula were prepared by suspending bacterial colonies from overnight growth cultures in 5 ml of sterile saline to match the turbidity of a 0.5 McFarland standard. A tenfold dilution was prepared for the inoculation of plates with a multipoint inoculator for a final inoculum of 10^4 ^colony forming units (CFU). Mueller-Hinton II and MacConkey agar plates were incubated at 37°C for 18–20 h.

Susceptibility testing by SEC plates was based on the instructions of manufacturer. Stock solution of each antimicrobial was diluted in distilled sterile water to obtain a final volume of 1 ml with anticipated working concentration of each antimicrobial. The 1 ml liquid volume was placed onto bottom film and stored for 2 h to allow the liquid to be absorbed. Then 2 μl of the *E. coli *inoculum (equivalent to 10^4 ^CFU) was spot-inoculated onto the hydrated film and left for 5–10 min before the top film was covered. Plates were incubated at 42°C for 18–20 h and growth appearing dark green to light blue-green indicated as *E. coli *according to the instructions of manufacturer.

For all of the three media, MIC was defined as the lowest concentration of an antimicrobial in a plate, which inhibited growth of the test strain. The reference strain *E. coli *ATCC 25922 was included in each experiment for quality control purpose (Table 1). All tests were done in duplicate.

### Data analysis

For each antimicrobial, MIC values were converted to Log_2 _MIC (mg/L) values to allow an overall description of the distribution of MIC values obtained by the three different media. Agreement between SEC plate and Mueller-Hinton II agar was defined as MIC values that differed by ± 1 log_2 _dilution or less in order to account for the inherent variation reading the results [12]. Using the breakpoints defined by CLSI [11] for *E. coli*, strains were classified as resistant, intermediate or susceptible, furthermore, cut off values according to EUCAST was presented (Table 1). SAS Version 9.1 was used to generate χ^2 ^tests to determine whether there were significant differences in the determination of resistance by the three media. Kappa statistics was used to determine the overall agreements in classification of strains based on their susceptibilities determined on SEC and Mueller-Hinton II agar plates [13].

## Results and discussion

In a pilot study with a range of strains belonging to *Enterobactericeae *and other bacterial species (including *E. coli*, *Salmonella, Shigella*, *Klebsiella, Citrobacter *and *Enterobacter*), we confirmed high specificity of SEC plates for isolation of *E. coli*, as only *E. coli *gave conspicuous growth on SEC plates (results not shown). SEC plate was designed for direct and simple quantitative detection of *E. coli*[8,9]. Typical *E. coli *on SEC plates appear as blue-green colonies as it has been found that about 97% of *E. coli *produce β-glucuronidase which reacts with an indicator dye in the plate media to produce dark green to blue-green colonies. Colonies other than *E. coli *are not conspicuous because they are colorless or have a light grey-beige color. The usefulness in using β-glucuronidase activity for identification of *E. coli *has been confirmed by Schraft and coworkers who reported almost identical colony counts based on β-glucuronidase activity and on classical biochemical reactions[6].

**Figure 1:**
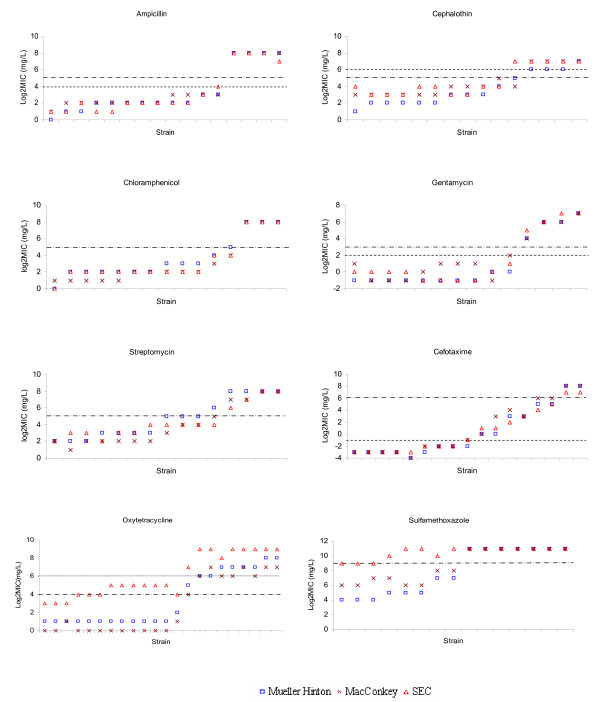
**Distribution of MIC (log_2 _mg/L) values for antimicrobial agents using Mueller Hinton II, MacConkey and SEC media**. -^.^-^.^- Break point values as defined by CLSI; ---- EUCAST epidemiological cut-offs; when only one line is shown, breakpoint and cut-offs are identical; ^........^ Adjusted breakpoint value for oxytetracycline.

All duplicates showed the same MICs except for three occasions (a difference by a 2-fold dilution), where the average MICs were used in the statistical analysis and the higher MIC values were used in Figure 1.

### MIC determinations for ampicillin, cephalothin, streptomycin, chloramphenicol, cefotaxime and gentamicin

Obtained MIC values for *E. coli *ATCC 25922 were within reference ranges in a consistent manner for all three media (Table 1). Figure 1 shows overall distributions of MIC (log_2 _mg/L) values for individual antimicrobial tested with the three media. MIC values obtained by individual medium showed a high degree of correlation with only minor differences seen for a few strains. The agreement of MIC values obtained by testing *E. coli *strains on SEC and Mueller-Hinton II agar plate media are shown in Table 2. Susceptibility testing with gentamicin, ampicillin, chloramphenicol and cefotaxime showed an almost perfect agreement between the two media (kappa values > 0.8) and a 100% agreement in values at the ± 1 log_2 _dilution level. Higher variations were observed for cephalothin and streptomycin with 73% and 87% agreement at the ± 1 log_2 _dilution level. However, agreements in MIC values as defined within ± 2 log_2 _dilutions error for test interpretation were 100% and 93%, respectively, and the kappa values (> 0.6) indicate substantial agreement for cephalothin and streptomycin MIC values obtained by the two media. A more than twofold difference in MIC values was noted for cephalothin in one stain, which changed its resistance category from susceptible to intermediate resistant (MIC values from 2 to 16 mg/L). Overall percentages of *E. coli *strains determined to be resistant to ampicillin, cephalothin, cefotaxime and gentamicin were identical for the two media. Compared to Mueller-Hinton II agar, the overall percentage of resistance on SEC plate were lower for chloramphenicol (20 versus 27%) and streptomycin (27 versus 33%), but not significantly lower (*P *> 0.05). This classified a few resistant *E. coli *strains into intermediate resistance at the ± 1 log_2 _dilution error level.

**Table 2:** Agreements of MIC values obtained by susceptibility testing *Escherichia coli *strains on SEC and Mueller Hinton II agar plate media.

Antimicrobial agent	No. of *E. coli*	Exact agreement		Within ± 1 dilution		Within ± 2 dilutions		Kappa value^a^
					
		No.	%	No.	%	No.	%	
Ampicillin	16	10	63	16	100	16	100	0.92
Cephalothin	15	4	17	11	73	14	93	0.67
Chloramphenicol	15	11	73	15	100	15	100	0.92
Gentamicin	14	7	50	14	100	14	100	0.93
Streptomycin	15	5	33	13	87	15	100	0.74
Cefotaxime	17	8	47	17	100	17	100	0.90
Oxytetracycline	22	0	0	3	14	11	50	0.48
Sulphamethoxazole	15	7	47	7	47	7	47	0.47

^a ^The kappa value indicates the level of agreement between MIC values obtained by SEC and Mueller Hinton II agar plate media as follows: < 0.2, slight agreement; 0.2–0.4, fair agreement; 0.4–0.6, moderate agreement; 0.6–0.8, substantial agreement; > 0.8, almost perfect agreement.

Overall, the good agreements between results obtained by the SEC and Mueller-Hinton II media in a diverse range of MICs to each of the six antimicrobials tested suggest that the SEC plate is suitable to discriminate resistant *E. coli *and non-resistant ones. The least agreement between MIC values obtained by SEC and Mueller-Hinton II media was by no more than ± 2 log_2 _dilution levels (except for one strain to cephalothin), an error level also seen when MIC values are read manually by different personnel and in repeated testing of identical strains [12].

### MIC determinations for oxytetracycline

*E. coli *ATCC 25922 demonstrated MIC values within the quality control range as shown on both the Mueller-Hinton II and MacConkey agar media, whereas, a higher MIC (8 mg/L) value was observed on SEC plate. Figure 1 showed a more than two log_2 _dilutions increase in MICs determined by SEC plates as compared with Mueller-Hinton II and MacConkey agar media (Figure 1). For these *E. coli *strains that were susceptible to oxytetracycline on Mueller-Hinton II agar (MIC = 2–4 mg/L), MICs obtained by SEC plates were 2–4 log_2 _dilutions higher (MIC = 8–32 mg/L). For the strains showing oxytetracycline resistance on Mueller-Hinton II agar (MIC = 32–256 mg/L), MICs obtained by SEC plates were 1–3 log_2 _dilutions higher (MIC = 128–512 mg/L). The overall agreement of MIC values obtained by Mueller-Hinton II agar and SEC plates was therefore moderate (kappa = 0.48) (Table 2). Exact agreement between MICs determined by the two media was 0 and agreement within ± 1 log_2 _dilution was only 14%. Overall percentage of strains determined as oxytetracyclin-resistant was significantly higher by SEC plates than that by Mueller-Hinton II agar (86 versus 41%; *P *< 0.01).

Mueller-Hinton II agar was originally developed specifically for antimicrobial susceptibility testing with a low concentration of calcium and magnesium; two substances that are known to reduce the activity of some antimicrobial agents, e.g. tetracycline [10]. In a previous study, a one to five log_2 _dilution increased in MICs of *E. coli *to tetracycline was observed when Mueller-Hinton broth was supplemented with 5 mg calcium and 2.5 mg magnesium per dl, confirming that tetracycline is bound to divalent cations, and therefore have reduced antibacterial activity [14]. This decreased antibacterial effect due to binding of oxytetracycline to divalent captions is a likely explanation of the increased MICs seen on SEC plates. Other factors, such as pH, would also affect the chemical binding and effect of oxytetracycline supplemented in SEC plate. Further studies are needed to determine the exact gradients of oxytetracycline concentrations and antimicrobial activity in SEC plate medium, as ingredient composition in SEC plate media is unknown. Also, the further studies are needed to assess if the lower kappa values shown by cephalothin and streptomycin could be associated with reduced antibacterial effects when these substances are added to the SEC medium.

Due to increased MIC values for oxytetracycline on SEC plates and difference in classification of strain susceptibility as compared with Mueller-Hinton II agar, an adjustment of MIC breakpoint for tetracycline would be needed if SEC plates are to be used for isolation of tetracycline-resistant *E. coli*. By increasing breakpoint value to 64 mg/L, the adjusted percentage of oxytetracycline resistance would be 86%, which was equal to the resistance percentage obtained on Mueller-Hinton II agar with a 16 mg/L breakpoint value. Likewise, overall kappa value for the two media would significantly improve to 0.73 when new breakpoint value was adopted. Therefore in order to confirm the new introduced breakpoint value for the SEC plates, an additional 20 randomly selected *E. coli *strains were tested for oxytetracycline susceptibility by Mueller-Hinton II agar and SEC plates (breakpoints of 16 mg/L and 64 mg/L were used individually). The eight susceptible strains with MIC values between 2–4 mg/L obtained on Mueller-Hinton II agar plate had MICs between 16–32 mg/L when tested on SEC plates, while the remaining 12 resistant *E. coli *strains with MICs between 32–128 mg/L on Mueller-Hinton II agar plate had MICs between 128–512 mg/L on SEC plates. These results showed a similar and consistent tendency in increasing MIC values on SEC plates and that with the adjusted breakpoint of 64 mg/L it is possible to classify resistant and susceptible strains correctly.

### MIC determination for sulphamethoxazole

*Escherichia coli *ATCC 25922 demonstrated MIC values within the quality control ranges shown on Mueller-Hinton II and MacConkey agars, however, the SEC plate gave an increased MIC values (1024 mg/L). A moderate overall agreement (kappa = 0.47) and an exact agreement of 47% on MIC values were observed for Mueller-Hinton II agar and SEC plate (Table 2). As MICs on SEC plates showed equal or larger than the upper limit of the test range (2048 mg/L) of sulphamethoxazole, this agreement level could possibly be decreased if a wider test range was used. However, sulphamethoxazole at concentrations higher than 2048 mg/L is not feasible to work with because of problems of dissolving the chemical at such high concentrations. The very high MIC values on SEC plates for sulphamethoxazole are likely caused by the well-known antagonism of ρ-aminobenzoic acid (PABA) on the activity of sulphonamides [15,16]. PABA naturally occurs in some tissue-based media but is also commonly added in media as a growth factor required by microorganisms. However, it is unknown whether and how much it is added to SEC plates. Mueller-Hinton II agar is widely recognized as the best suitable medium for sulfonamide resistance testing due to its very low content of PABA [10].

Performance of SEC plate in the study was highly reproducible with more than 95% duplicates giving the compatible results. The selected *E. coli *isolates used in this study covered diverse and broad ranges of MIC values to different antimicrobials allowing an evaluation of the overall distributions and agreements of MICs obtained by the selected media. In this study we chose to use CLSI breakpoints to classify strains as resistant, intermediate or susceptible in kappa analysis. Likewise, different definitions to discriminate resistant *E. coli *(e.g., cut-offs by EUCAST) with similar evaluation can be implemented and would likely result in new results of kappa analysis, but overall MIC distributions will be the same. For comparison purpose EUCAST cut-offs was included in Figure 1 and Table 1. When isolating or enumerating resistant *E. coli *from samples, a certain concentration of antimicrobial is needed to add in 1 ml inoculation sample suspension (or a serial dilution of suspension). After incubation, resistant *E. coli *grows conspicuous and colonies can easily be counted on a standard colony counter or manually. Because of a high specificity for *E. coli *as shown in previous studies and our pilot study, SEC plate eliminates needs for further identification tests of *E. coli*. As a result, the use of SEC plate for enumeration of antimicrobial resistant *E. coli *is much less labor-intensive and easier to perform compared to conventional selective agar-based methods, which provides a preferable option of *E. coli *selective media in antimicrobial resistance population study. It should be noted that SEC plate was not developed for antimicrobial susceptibility testing purposes and should not replace Mueller-Hinton II agar in MIC studies.

There were some limitations in this study, such as: only a limited number of isolates were used for each antimicrobial, and inoculums effect for enumeration was not evaluated, etc. A further study using SEC plate to isolate and enumerate resistant *E. coli *directly from samples would confirm its feasibility when handling bacteria population.

## Conclusion

This study provides the first information that the SEC plate medium is suitable to discriminate antimicrobial-resistant and non-resistant *E. coli *using CLSI standardized breakpoints. It appears feasible to use this medium to screen and enumerate resistant *E. coli *due to its high specificity and the good agreement obtained in MIC studies when compared with Mueller-Hinton II agar for most antimicrobials (ampicillin, cephalothin, streptomycin, chloramphenicol, cefotaxime and gentamycin). SEC plates may also be used to enumerate oxytetracycline-resistant *E. coli *if a plate-dependent breakpoint of 64 mg/L is used. However, SEC plate was not found suitable to select for sulphamethoxazole-resistant *E. coli*.

## Competing interests

The authors declare that they have no competing interests.

## Authors' contributions

WS performed the experiment and was responsible for the data analysis and writing the manuscript. EC participated in the study design and experimental work. AD and LJE were involved in the study design and revising the manuscript critically. All authors read and approved the final manuscript.
